# Comment on Hemostasis Using Prothrombin Complex Concentrate in Patients Undergoing Cardiac Surgery: Systematic Review with Meta-Analysis

**DOI:** 10.21470/1678-9741-2025-0302

**Published:** 2026-09-08

**Authors:** Stanislaw Vander Zwaag, Imre Kukel, Jens Fassl

**Affiliations:** 1 Institute of Cardiac Anesthesiology, TUD Dresden University of Technology, Dresden, Germany

Dear Editor,

We read with the utmost interest the meta-analysis published by Li et al. in your journal^[1]^. The safety and efficacy of prothrombin complex concentrate (PCC) remains a widely discussed topic, and the recent publication of the results of the FARES-II trial, the largest randomized controlled trial (RCT) to date, by Karkouti et al. in the Journal of the American Medical Association proved the usefulness of supplementation compared to fresh frozen plasma (FFP)^[2]^.

However, while preparing a scoping review on postoperative bleeding in cardiac surgery, we carefully read the abovementioned manuscript and would now like to pose several questions and comment on it.

Firstly, the authors analyzed “bleeding” and “thoracic drainage” as two separate outcomes in their meta-analysis. Most readers will not intuitively understand the difference between these two outcomes. As is common in cardiac surgery, shed blood from the operative field is usually reintroduced into the cardiopulmonary bypass or undergoes cell salvage; therefore, intraoperative bleeding is rarely reported as an outcome in trials. We hypothesized that the authors of the articles included in the meta-analysis might have used the terms “postoperative bleeding” and “thoracic drainage/chest tube output” interchangeably.

Six studies, including one RC T, reported “postoperative bleeding”, and five observational retrospective studies reported “thoracic drainage” as outcomes according to Li et al. However, reviewing the studies included in each sub-analysis reveals that three studies (Green et al., 2021; Harper et al., 2018; and Harris et al., 2020) were included in both sub-analyses (see Figure 1 for a direct comparison)^[3-5]^. In the case of Green et al., who reported the means and standard deviations directly in their manuscript, the values are the same in both sub-analyses. The minor differences in the values estimated from other studies may be due to the use of diferent methods to estimate means and standard deviations from manuscripts that report medians and interquartile ranges (IQR). The authors did not report the method (s) used for estimation, which prevented us from identifying the source of this issue.

**Fig. 1:**
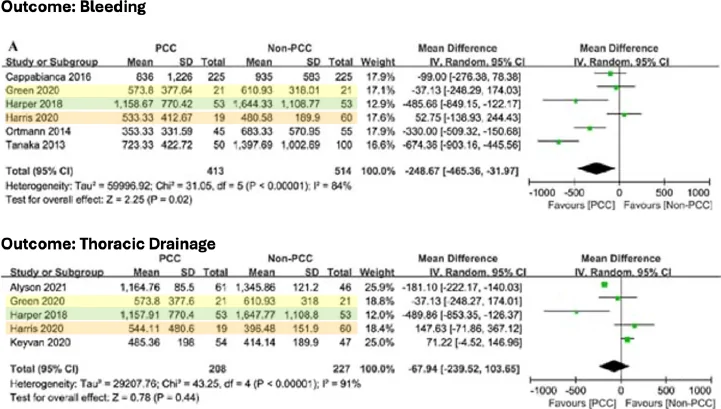
Analyses from the original article from Li et al., same studies with same or similar values are highlighted in yellow, green, and orange. CI=confidence interval; PCC=prothrombin complex concentrate; SD=standard deviation.

Secondly, in the case of two articles (Katz et al., 2021; Karkouti et al., 2021), the authors' names were reported as first names rather than last names (Alyson, 2021; Kevyan, 2020; respectively). Regarding Karkouti et al.'s study, Li et al. inverted the drainage output volumes (Karkouti et al. report a median of 450 ml [IQR 370–630] in the PCC group and 700 ml [IQR 470–950] in the FFP group, thus favoring PCC over FFP, contrary to the findings reported by Li et al).

Furthermore, we disagree with the grouping of FFP, recombinant factor VIIa (rFVIIa), and no intervention as a single control group to which PCC should be compared, as presented in the Li et al. manuscript. If this is the case, a subgroup analysis is required to evaluate the efficacy of the interventions. Such an analysis has already been performed in the excellent Cochrane review by Hayes et al. (2022)^[6]^.

To better reproduce the results of Li et al., we read the full texts of all the included studies in order to understand the reported outcomes. We found that, although the studies differed in their follow-up time (six studies reporting on 24-hour chest tube output and two studies followed up over 12 hours), they were indeed reporting on the same outcome. However, they used various definitions. Table 1 presents our findings.

**Table 1:** List of included studies with reported outcomes and quotes from the original articles pertaining to the outcomes.

Author	Year	Type	Reported outcome	Quote from the article / table	Type of PCC
Karkouti et al.^[9]^	2021	RCT	Chest tube output 24 h	Chest tube drainage, mL, 24 h	4-PCC
Cappabianca et al.^[10]^	2016	Obs	Chest tube output 24 h	24-hour postoperative blood loss	3-PCC
Green et al.^[3]^	2021	RCT	Chest tube output 24 h	Blood loss in chest drains at 24 h; ml	4-PCC
Harper et al.^[4]^	2018	Obs	Chest tube output 24 h	Chest tube output at 24 h, mL	3-PCC
Harris et al.^[5]^	2020	Obs	Chest tube output 24 h	Chest tube output, postoperative day 0 cumulative total, mL	4-PCC
Katz et al.^[11]^	2022	Obs	Chest tube output 24 h	Chest tube output, mL	4-PCC
Ortmann et al.^[12]^	2015	Obs	Chest tube output 12 h	Observed cumulative blood loss at (…) 12 h after ICU admission	4-PCC
Tanaka et al.^[13]^	2013	Obs	Chest tube output 12 h	12-hour CTD (mL)	3-PCC

CTD=chest thorax drainage; Obs=retrospective observational trial; 3-PCC=three-factor prothrombin complex concentrate; 4-PCC=four-factor prothrombin complex concentrate; RCT=randomized controlled trial

Furthermore, we performed a reanalysis of the included studies using corrected values from Karkouti et al. (2021) and including all studies in a subgroup analysis grouped by PCC type and comparator (FFP *vs.* rFVIIa *vs.* no PCC). We used Luo method to estimate means and standard deviations from the reported medians and IQR^[7]^. The results are presented in Figures 2 and 3. However, it is important to acknowledge that we did not conduct a systematic search or bias assessment, and we only included studies that were already included by Li et al. Consequently, our results cannot be regarded as confirmatory.

**Fig. 2:**
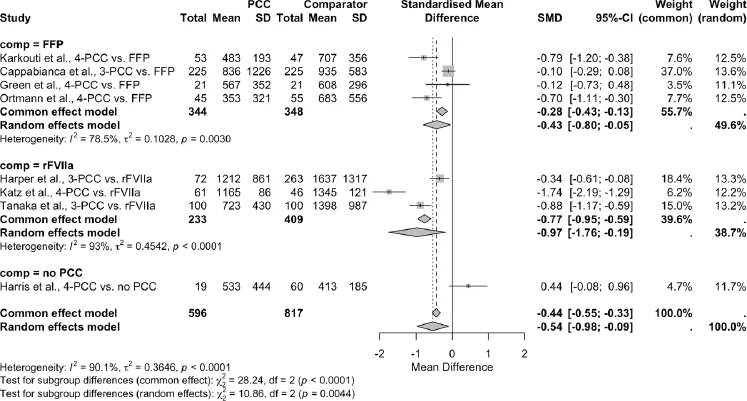
Results of meta-analysis with inclusion of all studies pertaining to the bleeding outcome, divided into subgroups based on the therapy in the control group. CI=confidence interval; FFP=fresh frozen plasma; PCC=prothrombin complex concentrate; rFVIIa=recombinant factor VIIa; SD=standard deviation; SMD=standardised mean difference.

**Fig. 3:**
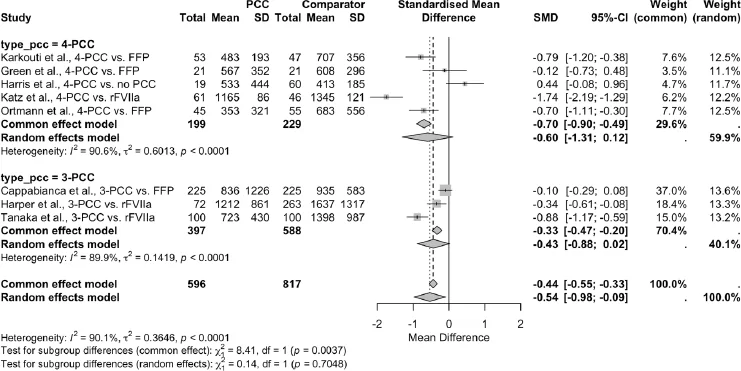
Results of meta-analysis with inclusion of all studies pertaining to the bleeding outcome, divided into subgroups based on the therapy in the intervention group. CI=confidence interval; FFP=fresh frozen plasma; PCC=prothrombin complex concentrate; rFVIIa=recombinant factor VIIa; SD=standard deviation; SMD=standardised mean difference.

As two of the studies reported on 12-hour output instead of 24-hour output, we used standardised mean differences instead of absolute mean differences. This was a pragmatic decision; however, it introduces an additional (yet already present in the original manuscript) heterogeneity. The source data as well as the R code for our analysis are available via open access on FigShare (DOI: 10.6084/ m9.figshare.29903249)^[8]^.

In summary, combining the studies into two different outcome groups in the meta-analysis by Li et al. yields more robust results that align with the outcomes of the recent study by Karkouti et al.^[2]^ Our results suggest that administration of PCC decreases the perioperative bleeding when compared with both FFP and rFVIIa. Future meta-analyses, including the results of FARES-II study, should further enhance the statistical significance and therefore clinical implications of the results.
